# Assessment of rewarming methods in unplanned out-of-hospital births from a prospective cohort

**DOI:** 10.1186/s13049-020-00750-9

**Published:** 2020-06-03

**Authors:** François Javaudin, Mélodie Roche, Lucile Trutt, Isabelle Bunker, Valérie Hamel, Sybille Goddet, François Templier, Christine Potiron, Quentin Le Bastard, Philippe Pes, Gilles Bagou, Jean-Louis Chabernaud, Emmanuel Montassier, Brice Leclère, on behalf of the AIE Group Investigators, Nathalie Laurent, Valérie Hamel, Dominique Foissin, Mickael Allouche, Claire Girardi, Hervé Degrange, Christelle Graf-Ammar, Magali Cotin, Thierry Debreux, Victor Tasteyre, Stéphane Meunier, Juliette Meunier, Adeline Sourbes, Vivien Brenckmann, Cyrielle Clape, Caroline Sanchez, Resa Dorostgou, Coralie Chassin, Sylvie Allard, Carole Bernard de Villeneuve, Régine Maupoint, Emilie Hue, Yacine Lamarche-Vadel, Solweig Barbier, Gaelle Le Bail, Katy Silverston, Jean-Louis Chabernaud, Fabrice Louvet, Eva Gallet, Valérie Demin, Nathalie Roudiak, Fatia Bouarfa, Catherine Ferrand, Sylvain Geoffroy, Bertrand Jestin, Cédric Gangloff, Adelaide Denoel, Julien Miklin, Stéphane Chateaux, Sylvain Ambard, Yoann Evain, Christine Goubet-Potiron, Hélène Broch, Pierre Houdayer, Lucile Bruere-Ronzi, Caroline Savatier, Elsa Rocour, Bruno Rohee, Guillaume Barre, Dominique Chevalier, Mohamed Touil, Juliette Foucher, Sylvie Baumard, Frédéric Saura, Christine Jaulin, Hélène Bellanger, Romain Cheyssac, Caroline Jimenez, Chloe Carruesco, Marianne Corbillon, Delphine Garnier, Marie-Laure Devaud, Anne-Sophie Pruliere, Nathalie Laurent, Aurélie Guinard, Hervé Degrange, Jean-Claude Lecuit, Anne-Sophie Lucas

**Affiliations:** 1 Department of Emergency Medicine, University Hospital of Nantes, Nantes, France; 2 MiHAR lab, University of Nantes, Nantes, France; 3 Department of Emergency Medicine, District Hospital Centre, La Roche-sur-Yon, France; 4 Department of Medical Evaluation and Epidemiology, University Hospital of Nantes, Nantes, France; 5 Pediatric Intensive Care Unit, University Hospital of Nantes, Nantes, France; 6 Department of Emergency Medicine, Toulouse Purpan University Hospital, Toulouse, France; 7 Department of Emergency Medicine, University Hospital of Dijon, Dijon, France; 8 Department of Emergency Medicine, University Hospital of Angers, Angers, France; 9 Department of Emergency Medicine, University Hospital of Lyon, Lyon, France; 10 Neonatal Emergency Transport Team, SAMU 92, Neonatal Intensive Care Unit, South-Paris University Hospitals (AP-HP), A. Béclère Clamart University Hospital, Paris, France

**Keywords:** Unplanned out-of-hospital birth, Rewarming method, Neonatology

## Abstract

**Background:**

Mobile intensive care units frequently manage unplanned out-of-hospital births (UOHB). Rewarming methods during pre-hospital management of UOHB have not yet been compared. The aim was to compare rewarming methods used during pre-hospital management in a large prospective cohort of UOHB in France.

**Methods:**

We analysed UOHB from the prospective AIE cohort from 25 prehospital emergency medical services in France. The primary outcome was the change in body temperature from arrival at scene to arrival at hospital.

**Results:**

From 2011 to 2018, 1854 UOHB were recorded, of whom 520 were analysed. We found that using incubator care was the most effective rewarming method (+ 0.8 °C during transport), followed by the combination of plastic bag, skin-to-skin and cap (+ 0.2 °C). The associations plastic bag + cap and skin-to-skin + cap did not allow the newborn to be warmed up but rather to maintain initial temperature (+ 0.0 °C). The results of the multivariate model were consistent with these observations, with better rewarming with the use of an incubator. We also identified circumstances of increased risk of hypothermia according to classification and regression tree, like premature birth (< 37 weeks of gestation) and/or low outside temperature (< 8.4 °C).

**Conclusions:**

Using an incubator was the most effective rewarming method during pre-hospital management of UOHB in our French prospective cohort. Based on our model, in cases of term less than 37 weeks of gestation or between 37 and 40 weeks with a low outside temperature or initial hypothermia, using such a method would be preferred.

## Background

The prevalence of unplanned out-of-hospital births (UOHB) is estimated to represent 0.6% of all deliveries in the United States, 1 to 2% in the UK and 0.5% in France [1, 2]. UOHB are defined as births without midwife and medical care, or without optimal health care conditions [3]. This specific context must be discriminated from planned out-of-hospital births, home births or freestanding birthing centers, where midwife management is performed [4].

Out-of-hospital delivery is associated with unfavorable perinatal outcomes and increased mortality [2, 5, 6], with hypothermia being the most frequently described adverse outcome [1, 7, 8]. Indeed, hypothermia is recognized as a significant risk factor for mortality under these conditions [9, 10]. In low birth weight infants, mortality increases by 28% per 1 °C decrease of body temperature from birth to admission in the neonatal intensive care unit [11].

Many in-hospital studies have evaluated rewarming methods, including incubator care, skin-to-skin contact, and plastic wrap [12–19]. Current guidelines suggest using plastic wraps or skin-to-skin contact to maintain the temperature of the newborn during the first hour in resource-limited settings [20]. However, no study has compared the efficacy of these methods out of hospitals.

The aim of our study was to compare rewarming methods used during pre-hospital management in a large prospective multicentric cohort of UOHB in France.

## Methods

### Setting and Design of the Study

We analysed data from the prospective multicenter cohort of unplanned out-of-hospital births named AIE [21] (observatoire des Accouchements Inopinés Extra-hospitaliers: out-of-hospital unexpected deliveries cohort). The AIE cohort involved 25 of the 103 prehospital emergency medical service (EMS) units in France. The EMS units only receive medical or trauma calls. Following protocols, the first dispatcher obtains the basic information from the caller, and then transfers the call to an emergency physician dispatcher, who performs a medical evaluation and decides the appropriate level of emergency medical response [22]. These units are also ambulance base stations equipped with one or more mobile intensive care units (MICU), consisting of an ambulance driver, a nurse, and a senior emergency physician as minimum team. The data from this cohort were collected by the emergency physician of the MICU who managed the UOHB, then verified and stored on an online secured server. All UOHB managed by a MICU were included in the database. The criteria for non-inclusion were: a birth in hospital and lack of maternal consent. The choice of rewarming method was left to the discretion of the emergency physician and the devices available during the prehospital management.

Our database was approved by the French Data Protection Authority (CNIL n°9,112,033 and CCTIRS) and by a French research ethics committee. Maternal consent was systematically requested after birth management.

### Data collection and inclusion criteria

Each UOHB managed by a MICU of the participating centers was recorded in the AIE database. From this AIE database, we included newborns with a body temperature measured i) at MICU arrival on scene and ii) at MICU arrival at hospital. Meteorological data (outdoor temperature) were obtained retrospectively for each UOHB from the French national meteorological service (Météo France) database (for place and time) [23]. Hypothermia was defined as a body temperature < 36.5 °C. Hypothermia between 36 °C and 36.4 °C was considered mild, < 36 °C moderate and < 32 °C severe, according to the definitions set out by the World Health Organization [24]. Hyperthermia was defined as a body temperature > 37.5 °C [24].

### Outcome of the study

The primary outcome was the change in body temperature from MICU arrival on the scene to arrival at the hospital, according to the rewarming methods employed by the MICU.

### Statistical analysis

Data were collected in a secure database and then extracted in the form of a spreadsheet that was processed using Excel software (Microsoft Systems, Redmond, Washington, USA). Subjects with missing data for variables of interest were excluded from the analysis of the primary outcome. Continuous variables are presented as the median and the interquartile range (IQR) in parenthesis. Categorical variables are summarized by patient counts and percentages. Chi-square, Mann–Whitney U and Kruskal–Wallis tests were used to compare groups when appropriate. The efficacy of the rewarming methods was measured by the difference in body temperature of the newborn between MICU arrival at the scene and arrival at the hospital. An adjusted comparison of the rewarming methods was performed using a multivariate linear regression, based on variables selected via the lasso method. Classification and Regression Tree (CART) analysis was also performed to identify circumstances at increased risk of hypothermia. *P*-values lower than 0.05 were considered significant. The statistical analyses were performed using the R software version 3.5.1.

## Results

### Baseline characteristics of the study population

**Fig. 1:**
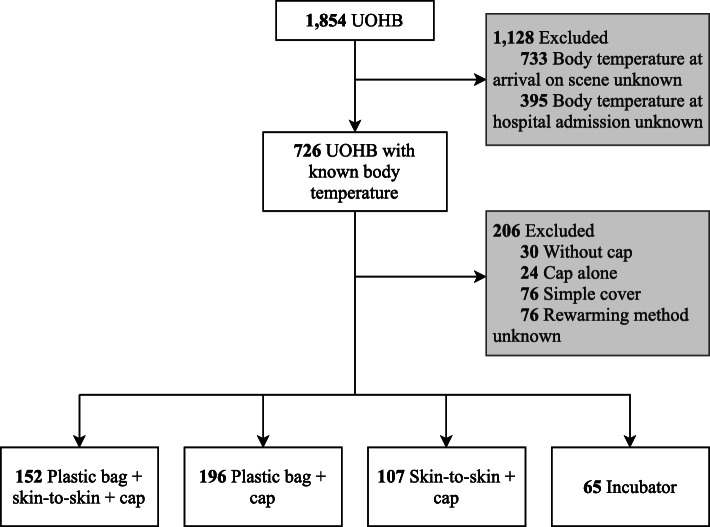
Study flow chart. UOHB, unplanned out-of-hospital births

From October 2011 to January 2018, a total of 1854 UOHB were recorded from 25 prehospital emergency medical service units in France. Among these, 733 (40%) newborns did not have body temperature measurement on arrival at the scene, and 395 (21%) at hospital admission. Therefore, we finally included data from 726 (39%) newborns (Fig. 1).

Most UOHB occurred at home (*n* = 586; 81%), and women mainly delivered before the arrival of mobile intensive care units (*n* = 502; 69%). The median term was 40 weeks of gestation (IQR: 39–41) and the median newborn weight was 3200 g (IQR: 2900–3523). These parameters were not statistically different in the 1128 excluded UOHB: the median gestational age was 40 weeks of gestation (IQR: 39–41; *P* = 0.63) and the median birth weight was 3170 g (IQR: 2900–3480; *P* = 0.06). The median outdoor temperature was 11.9 °C (IQR: 7.2–17.6; range: − 5.9 °C to 33.8 °C). The median of the on-scene body temperature was 36.0 °C (IQR, 35.4–36.7), measured by the MICU with a median delay of 20 min after delivery (IQR: 10–30), mainly by the rectal route (*n* = 436; 60%). On hospital admission, 321 (44%) newborns did not have hypothermia (i.e., body temperature > 36.4 °C), 206 (28%) had mild hypothermia (i.e., between 36.0 to 36.4 °C) and 199 (27%) had moderate to severe hypothermia (i.e., < 36.0 °C). Only one newborn was severely hypothermic (< 32 °C) on admission to the hospital. The different characteristics of UOHB, depending on their thermal status, are presented in the Table 1. We counted 9 newborns with hyperthermia (> 37.5 °C), once they arrived at the hospital; of these, 2 already had hyperthermia on the scene. The median body temperature at hospital admission was 36.3 °C (IQR: 35.8–36.8). During the pre-hospital phase, skin-to-skin contact was made for 310 (43%) newborn infants, and plastic bags, caps and incubators were used respectively for 418 (58%), 665 (92%) and 72 (10%) newborn infants.

**Table 1:** Population Characteristics

**Variables**	**Normothermia at hospital admission** (> 36,4 °C) (*n* = 321)	**Mild Hypothermia at hospital admission** (36–36,4 °C) (*n* = 206)	**Moderate or Severe Hypothermia at hospital admission** (< 36 °C) (*n* = 199)	***P***	**Number of missing or unknown data, n (%)**
Delivery before medical team arrival, n (%)	200 (62)	140 (68)	162 (81)	< .001	0 (0)
Place of delivery, n (%)				.004	0 (0)
Public place	33 (10)	12 (6)	22 (11)		
Home or private place	243 (76)	177 (86)	166 (83)		
Ambulance	45 (14)	17 (8)	11 (6)		
Outside temperature, median (IQR), °C	13.5 (8.5–19.6)	12.4 (7.4–17.8)	9.6 (5.7–14.3)	< .001	0 (0)
Maternal age, median (IQR), y	31 (28–35)	31 (27–34)	31 (27–34)	.4	1 (< 1)
Weeks of gestation, median (IQR), w	40 (39–41)	40 (39–41)	39 (39–40)	< .001	43 (6)
Good adaptation to extrauterine life*, n (%)	313 (97)	200 (97)	187 (94)	.09	0 (0)
Gender male, n (%)	163 (51)	93 (45)	85 (43)	.2	0 (0)
Weight, median (IQR), g	3300 (3010–3620)	3220 (2980–3480)	3000 (2675–3340)	< .001	25 (3)
Body temperature at arrival on scene, median (IQR), °C	36.5 (36.0–37.0)	36.0 (35.5–36.7)	35.4 (35.0–36.0)	< .001	0 (0)
Body temperature site at arrival on scene, n (%)				.7	73 (10)
Rectal	183 (57)	123 (60)	130 (65)		
Axillary	87 (27)	47 (23)	45 (23)		
Other	20 (2)	12 (6)	12 (6)		
Time from delivery to initial temperature, median (IQR), min	18 (9–30)	16 (8–29)	22 (10–32)	.01	103 (14)
Time from delivery to hospital admission, median (IQR), min	45 (35–60)	40 (30–55)	40 (30–51)	.003	281 (39)
Body temperature site at hospital admission, n (%)				.2	194 (27)
Rectal	176 (55)	115 (56)	106 (53)		
Axillary	58 (18)	31 (15)	39 (20)		
Other	5 (2)	5 (2)	1 (1)		
Rewarming methods (in isolation), n (%)					9 (1)
Skin-to-skin	137 (43)	87 (42)	86 (43)	.9	
Plastic bag	188 (59)	98 (48)	132 (66)	< .001	
Cap	301 (94)	182 (88)	182 (91)	.09	
Incubator	35 (11)	22 (11)	15 (7)	.4	
Mortality, n (%)	0 (0)	1 (1)	0 (0)	.6	2 (< 1)

*IQR* interquartile range

* Apgar scores of 7 to 10

### Assessment of rewarming methods

We assessed all possible combinations of newborn warming methods reported in the database: plastic bag + skin-to-skin + cap (*n* = 152; 21%, reference), plastic bag + cap (*n* = 196, 27%), skin-to-skin + cap (*n* = 107, 15%) and incubator (*n* = 65, 9%). Importantly, the other combinations (*n* = 130, 18%) (i.e., without cap, cap alone or no specific methods such as a simple cover) were not analysed because the subgroups were too small or not effective enough. Moreover, missing or unknown data were also found in 76 subjects (10%), leaving 520 subjects for primary outcome analysis (Fig. 1).

**Fig. 2:**
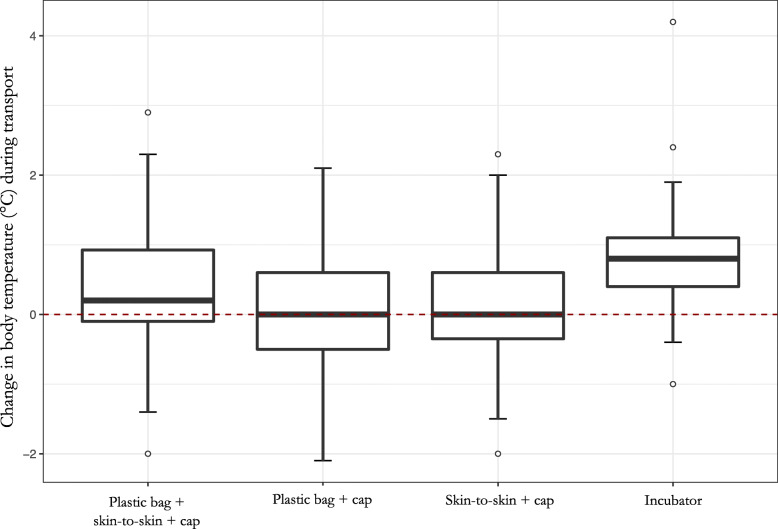
Change in body temperature during transport according to the rewarming methods

We showed that using an incubator was the most effective rewarming method in the pre-hospital phase (*P* < .001), with a median temperature difference of + 0.8 °C between on scene arrival and hospital admission. Comparatively, the reference association (i.e., plastic bag + cap + skin-to-skin) was associated with a median increase of + 0.2 °C while the other methods only maintained body temperature without rewarming (median difference of 0.0 °C) (Fig. 2). Indeed, between on-scene arrival and hospital admission, the proportion of normothermic newborns increased from 20 to 49% with an incubator, from 32 to 49% with the reference method, from 38 to 43% with the plastic bag + cap combination, and from 43 to 37% with the skin-to-skin + cap combination (Figure S1 in Supplementary Material). Concerning the 9 subjects with hyperthermia, 3 of them were rewarmed up by the combination of plastic bag + skin-to-skin + cap, 2 by plastic bag + cap, 2 by skin-to-skin + cap and 2 by incubator.

Based on the multivariate linear model, predictive factors associated with a significant change in body temperature were outside temperature, weeks of gestation, weight and first body temperature measured (Table 2). We also found that the most effective rewarming method was the use of an incubator. The plastic bag + cap association was less effective than plastic bag + cap + skin-to-skin combination (reference), with an adjusted temperature difference (difference between final temperature at hospital and initial on scene of UOHB) of − 0.18 °C (95CI, − 0.32; − 0.05). The skin-to-skin + cap combination was associated with a change in body temperature of − 0.15 °C (95CI, − 0.31; 0.01) compared to the reference (*P* = 0.07). In order to facilitate the interpretation of Table 2, here is an example: the adjusted temperature difference increased by + 0.16 °C when the outside temperature increased by + 10 °C. The weight of the newborn was positively associated with rewarming during transport, the adjusted temperature gain was + 0.25 °C per kilogram. Indeed, the heavier the newborn weighed, the warmer it got during transport. By analyzing in subgroups with the same model, we found that in the initially hypothermic population (body temperature ≤ 36.4 °C) at the birth site, the incubator was better than other rewarming methods (Table S1 in Supplementary Material). Among the normothermic population, the plastic bag + cap + skin-to-skin combination was more effective than skin-to-skin + cap and plastic bag + cap, and seemed to be as efficient as the incubator (Table S2 in Supplementary Material). Median transfer time to hospital was 38 min (IQR: 31–49) in the incubator group, 45 min (IQR: 24–66) in the skin-to-skin + cap group, 45 min (IQR: 34–55) in the plastic bag + cap group, and 43 min (IQR: 33–60) in the plastic bag + cap + skin-to-skin group (*P* = 0.02; 39% of missing data).

**Table 2:** Multivariate linear model of factors associated with change in body temperature during transport

**Variables**	**Adjusted temperature difference, °C (95% CI)**
**Outside temperature (per 10 °C)**	0.16 (0.08; 0.24)
**Weeks of gestation**	0.04 (0.003; 0.08)
**Weight (per kg)**	0.25 (0.10; 0.39)
**Rewarming methods**	
Plastic bag + cap + skin-to-skin	reference
Plastic bag + cap	−0.18 (−0.32; −0.05)
Skin-to-skin + cap	−0.15 (−0.31; 0.01)
Incubator	0.33 (0.13; 0.52)
**Initial body temperature (per °C)**	−0.53 (− 0.59; − 0.46)

*95% CI* 95% confidence interval

### Identification of circumstances with increased risk of moderate to severe hypothermia

**Fig. 3:**
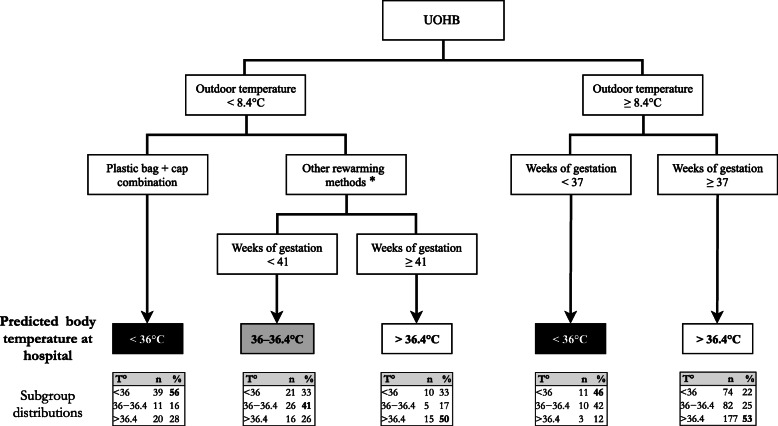
Classification tree to predict the body temperature of the newborn at hospital admission after unplanned out-of-hospital birth. * Other rewarming methods, plastic bag + skin-to-skin + cap combination, skin-to-skin + cap combination or incubator. UOHB, unplanned out-of-hospital birth. This tree was constructed using the CART method (Classification and Regression Trees). For example, in UOHB if the outside temperature is ≥8.4 °C and the gestation term is less than 37 weeks, the predicted body temperature of the newborn on arrival at the hospital is < 36 °C

We identified 3 circumstances with an increased risk of hypothermia according to the CART method (Fig. 3). First, when the outdoor temperature was ≥8.4 °C and the term was less than 37 weeks of gestation, the probability of hypothermia was 88%. Second, when the outdoor temperature was < 8.4 °C and the rewarming method was the plastic bag + cap combination, the probability of hypothermia was 72%. Third, when the outdoor temperature was < 8.4 °C and another rewarming combination other than the plastic bag + cap combination was used with a term between 37 and 40 weeks of gestation the probability of hypothermia was 74% (Fig. 3).

## Discussion

To the best of our knowledge, this is the first study that has assessed rewarming methods in a large, prospective and multicenter cohort of UOHB. We found that the incubator was the most effective method but also that the combination of plastic bag + cap + skin-to-skin seems to be a useful alternative in most cases.

The definition of hypothermia differs from one study to another. For those applying a cut-off of < 36.5 °C, the prevalence of UOHB hypothermia ranged from 30 to 100% [25, 26]. We also used this definition, and found a prevalence of hyperthermia of 56%. Others defined hypothermia with a cut-off of < 35 °C or < 35.5 °C and found the proportion of hypothermic newborns to be between 29 and 60% [7, 27]. In premature (24–35 weeks of gestation) newborns, Jones et al. regained an average temperature of 33.3 °C in the event of UOHB [9]. Although the prevalence of hypothermia varies from study to study, our estimate is nevertheless consistent within these heterogeneities.

Obviously hypothermia is common in UOHB, but it is also common in hospital-born infants (32 to 85%) [12]. Previous studies reported that skin-to-skin is an effective warming method as compared to the incubator for hospital births in both premature and low-risk newborns [13–15]. The polyethylene plastic bag/wrap is also considered a safe and effective method for term and premature newborns in those circumstances [28, 29]. In our study of UOHB, we showed that the combination of these different methods facilitated the rewarming of newborns, but that overall, the incubator was more efficient.

We also found that a low outside temperature was associated with an increased risk of hypothermia in UOHB. This finding is coherent with the results of previous studies. For example, Mullany et al. reported an increased risk of hypothermia in at-term newborn infants during the cold season in Nepal [30]. This same association was also described in West Africa and in Italy [31, 32]. Using outside temperature as a continuous variable, we were able to define a threshold of increased risk of hypothermia, namely below 8.4 °C. In this circumstance of increased risk of hypothermia, or when the baby is premature (less than 37 weeks of gestation), we recommend using the most efficient rewarming method for the pre-hospital phase, which seems to be incubator in our study. Indeed, in intra-hospital conditions, servo-controlled incubators with skin temperature set at 36.5 °C decrease neonatal mortality [33]. During the initial stabilization of very premature babies before retrieval by a neonatal emergency transport team, several interventions should be combined: woolen or plastic caps, polyethylene bag/wrap and a radiant, servo-controlled transport incubator [9, 16, 19, 20, 33]. In France, since the late 70’s, neonatal transfers have been carried out by specialized teams with MICU including a consultant paediatrician [9, 16, 34, 35]. These teams play a critical role in prehospital newborn stabilization and transportation in cases of high-risk UOHB [35, 36]. Warming and humidification of gases used, when giving respiratory support to preterm infants for stabilization at birth and transfer to the neonatal intensive care unit (NICU), may also improve temperature [ 37–39]. But it is only possible if the MICU is equipped with a continuous heat source powered by portable battery [35–37]. Moreover, the cost of a transport incubator is currently between $2000 and $8000. These bulky devices take up space in ambulances. If only a few ambulances are equipped, the risk is that the time to arrive on the scene will be longer because they would not be the closest ambulance; which would most likely result in aggravating hypothermia. Perhaps less voluminous and less expensive collapsible or inflatable transport equipment could solve this problem. Indeed, when the delivery took place before the arrival of the medical team, the risk of hypothermia was more important. This is why it is necessary to reduce the time to arrive on site as much as possible. For example, we observed that children born in ambulances, i.e. in the presence of professional rescuers, were more often normothermic at hospital admission while those born at home were more at risk of hypothermia. Indeed, the time to reach the hospital was shorter and the medical team will take charge immediately in this case. As a result, the newborn could not cool down much under these conditions. Moreover, it was also found that if the UOHB took place before the arrival of the medical team, regardless of the place of delivery, the newborn was more likely to be hypothermic. In this situation, the rewarming methods used by non-professionals seem to be ineffective in maintaining the newborn body temperature. Indeed, when delivery was more frequent before the arrival of the medical team, the initial body temperature of the newborn at arrival on scene was lower.

Quality improvement initiatives, including staff training, use of checklists and continuous feedback with the staff involved in the prehospital management of the neonate are key factors to prevent heat loss from the scene of birth to admission to the NICU [34].

### Limitations

The main limitation of our study was that although AIE data are very complete for some variables, missing data and use of null values occur more often for others. Indeed, 61% of the initial or final temperatures were not measured or reported, which excluded many subjects from our study. This high rate of missing data may have caused a selection bias and had an impact on the statistical power of our study. Other variables were also misinformed, such as intervention times. For example, we noticed that the time from delivery to hospital admission was longer in the normothermia group at hospital admission. This can be explained by the fact that this group benefited from more efficient warming than the others and that the more time they spent in the transfer the more they warmed up. But due to 39% missing data we couldn’t interpret it correctly (same thing with the transfer to hospital times according to the warming method).

Another limitation was the heterogeneity of the site for measuring the body temperature of newborns. This deviation may have led, for our primary outcome, to false increases and decreases in the difference between on scene and hospital admission when measured in different ways. However, these discrepancies were probably relatively small: indeed, a systematic review on the comparison between rectal and axillary temperature measurements in newborns concluded that the average difference was + 0.17 °C (95CI, − 0.15 to 0.50) [40].

Finally, as our study was not randomized, the estimations of the efficacy of each rewarming method might be biased by confounding factors. We tried to limit confounding by adjusting on several variables in our models, but it is possible that some clinical or environmental factors that might have influenced both the choice of the rewarming method and the final outcome were not considered.

## Conclusions

This study was the first to assess rewarming methods in a large and multicenter cohort of unplanned out-of-hospital births. We found that the transport incubator was the most effective method, but a combination of plastic bag + skin-to-skin + cap seems useful in most cases if no mobile incubator is available. However, in case of term < 37 weeks of gestation (premature birth) or between 37 and 40 weeks (full term babies) and a low outside temperature (< 8 °C) or initial hypothermia, we recommend the use of an incubator. Future studies are required to investigate strategies to optimize the management of newborns in the prehospital area.

## Supplementary information

**Additional file 1: Figure S1.** Evolution of the distribution of newborns according to their initial and final body temperature. **Table S1.** Multivariate linear model of factors associated with change in body temperature during transport. **Table S2.** Multivariate linear model of factors associated with change in body temperature during transport.

## Data Availability

The datasets used and analysed during the current study are available from the corresponding author on reasonable request.

## Abbreviations

UOHB: Unplanned out-of-hospital births
AIE: Observatoire des Accouchements Inopinés Extra-hospitaliers: out-of-hospital unexpected deliveries cohort
EMS: Emergency medical service
MICU: Mobile intensive care units
IQR: Interquartile range
CART: Classification and Regression Tree
NICU: Neonatal intensive care unit
