# Supplementary material for: Assessment of rewarming methods in unplanned out-of-hospital births from a prospective cohort
Source: Scand J Trauma Resusc Emerg Med. 2020 Jun 3;28:50. doi: 10.1186/s13049-020-00750-9 (PMC7271438; doi:10.1186/s13049-020-00750-9)
Supplement: Supplementary file 1 — Additional file 1: Figure S1. Evolution of the distribution of newborns according to their initial and final body temperature. Table S1. Multivariate linear model of factors associated with change in body temperature during transport. Table S2. Multivariate linear model of factors associated with change in body temperature during transport. [file 13049_2020_750_MOESM1_ESM.docx]

**Supplementary Material**

**Figure S1.** Evolution of the distribution of newborns according to their initial and final body temperature

Initial T°C, body temperature on the scene; Final T°C, body temperature at hospital.

**Table S1.** Multivariate linear model of factors associated with change in body temperature during transport

| **Variables** | **Adjusted temperature difference, °C (95% CI)** |
| --- | --- |
| **Outside temperature (per 10°C)** | 0.16 (0.05; 0.28) |
| **Weeks of gestation** | 0.05 (0.002; 0.09) |
| **Weight (per kg)** | 0.22 (0.05; 0.39) |
| **Rewarming methods** |  |
| Plastic bag + cap + skin-to-skin | reference |
| Plastic bag + cap | -0.17 (-0.35; 0.01) |
| Skin-to-skin + cap | -0.09 (-0.31; 0.13) |
| Incubator | 0.35 (0.11; 0.60) |
| **Initial body temperature (per °C)** | -0.51 (-0.61; -0.41) |

95% CI, 95% confidence interval

**Table S2.** Multivariate linear model of factors associated with change in body temperature during transport

| **Variables** | **Adjusted temperature difference, °C (95% CI)** |
| --- | --- |
| **Outside temperature (per 10°C)** | 0.16 (0.05; 0.28) |
| **Weeks of gestation** | 0.01 (-0.05; 0.08) |
| **Weight (per kg)** | 0.21 (0.04; 0.39) |
| **Rewarming methods** |  |
| Plastic bag + cap + skin-to-skin | reference |
| Plastic bag + cap | -0.22 (-0.43; -0.01) |
| Skin-to-skin + cap | -0.25 (-0.48; -0.02) |
| Incubator | 0.24 (-0.12; 0.60) |
| **Initial body temperature (per °C)** | -0.57 (-0.83; -0.32) |

95% CI, 95% confidence interval
